# Field Survey and Resistance Occurrence to ALS-Inhibiting Herbicides in *Glebionis coronaria* L. in Tunisian Wheat Crops

**DOI:** 10.3390/plants9091210

**Published:** 2020-09-16

**Authors:** Zeineb Hada, Messaad Khammassi, Houda Jenfaoui, Yosra Menchari, Joel Torra, Thouraya Souissi

**Affiliations:** 1 Department of Plant Health and Environment, National Agronomic Institute of Tunisia, University of Carthage, Tunis 1082, Tunisia; jenfhouda@gmail.com; 2 Laboratory of Bioagressor and Integrated Management in Agriculture (LR14AGR02), National Agronomic Institute of Tunisia, University of Carthage, Tunis 1082, Tunisia; kh_messad@yahoo.fr (M.K.); menchariyosra@yahoo.fr (Y.M.); 3 National Institute of Field Crops, Boussalem, Jendouba 8170, Tunisia; 4 Higher Institute of Biotechnology of Beja, University of Jendouba, Jendouba 9000, Tunisia; 5 Department of Hortofructicultura, Botànica i Jardineria, Agrotecnio, Universitat de Lleida, 25198 Lleida, Spain; joel.torra@udl.cat

**Keywords:** *Glebionis coronaria* (L.) Cass. ex Spach, durum wheat, herbicide resistance, mesosulfuron + iodosulfuron, tribenuron-methyl

## Abstract

*Glebionis coronaria* (L.) Cass. ex Spach is a troublesome weed in cereal cropping systems in northern Tunisia. Recently, failures in controlling this weed have been reported by farmers. Field surveys and farmers interviews were conducted to highlight the potential causes of *G. coronaria* occurrence and the associated yield losses in wheat. Survey results revealed a significant correlation between farmers’ awareness of resistance occurrence and cultural practices, mainly sowing date and tillage, while *G. coronaria* abundance was related to the lack of herbicide rotation and the frequency of ALS-inhibiting herbicide use. High *G. coronaria* infestations (more than 20 plants/m^2^) caused a significant decrease in wheat grain yield, reaching almost 75% at a density of 100 plants/m^2^. Field and pot experiments showed low efficacies of ALS-inhibiting herbicides to control *G. coronaria* populations. The application of field rates of tribenuron-methyl and mesosulfuron + iodosulfuron failed to control the tested populations, and generally, *G. coronaria* dry weight increased compared to nontreated ones (potential hormetic effect). These findings were further investigated in two selected resistant populations through tribenuron dose–response experiments, plants from both populations exhibited high resistance factors (greater than 300), surviving up to 16-fold the recommended field dose. This is the first report for *G. coronaria* resistance occurrence to ALS-inhibiting herbicides in Tunisia and the second case worldwide.

## 1. Introduction

*Glebionis coronaria* (L.) Cass. ex Spach, known also under the common name of crown daisy or chrysanthemum, is an annual plant belonging to the Asteraceae family. It is frequent as ruderal vegetation, in field margins, road verges, and urban wastelands, and as a weed in several crops [1]. In Tunisia, *G. coronaria* is common in all climatic stages, being more abundant in the humid and the subhumid areas and at high infestation levels, competing vigorously with cereals and other annual crops for space and nutrients [2]. Cereals are sown in 1.5 million hectares (i.e., one third of the Tunisian arable land) under rained conditions, predominantly, in the Northern and Western parts of the country. Durum wheat is the most widely cultivated cereal in Tunisia, representing 54% of total cereals [3]. However, yields fluctuate from one year to another mainly due to abiotic and biotic stresses and weeds constitute one of the major technical and economic limitations of wheat production [4].

Tunisian farmers heavily rely upon acetolactate synthase (ALS) inhibitors for weed management in cereal fields. These herbicides target the activity of ALS enzyme in weeds, which is the first enzyme involved in the biosynthetic pathway of branched-chain amino acids (leucine, isoleucine, and valine), resulting in a rapid growth cessation in susceptible species by inhibiting the enzyme activity [5,6]. ALS-inhibiting herbicides have been extensively applied worldwide due to their high efficacy, environmental safety, minimal mammalian toxicity, low cost, wide crop selectivity, and broad spectrum weed control at very low application rates [7]. Consequently, the overuse of ALS-inhibiting herbicides has resulted in rapid weed resistance evolution compared with other groups of herbicides [6,8], resulting in 792 cases of resistance belonging to 165 species around the world [9]. Generally, the herbicide resistance evolution is widely influenced by the cultural and management practices [10] and the main drivers are high weed densities, repeated use of the herbicides that have the same mode of action (MoA), tillage practices, or crop monoculture, among others [11,12,13,14,15]. With the aim to delay the evolution of herbicide resistance, the recommendations have mainly focused on guidelines of herbicide application such as herbicide rotation and herbicide mixtures [16]. Grower’s awareness is one of the major key factors that delays the occurrence of herbicide-resistant weeds, attributed to the primordial role of growers in keeping field records, when weed resistance is suspected, for future herbicide monitoring [10].

Recent observations in winter cereals throughout the Northern regions in Tunisia revealed that *G. coronaria* is frequently present and farmers failed to control it in wheat cropping systems, especially in the Bizerte region, which raises suspicion of the occurrence of herbicide resistance. The only case known worldwide of *G. coronaria* with confirmed resistance to ALS-inhibiting herbicides was reported in 2004 [17]. To date, despite the wide distribution of *G. coranaria*, there are almost no studies quantifying the geographical extent, severity, and level of herbicide resistance in Tunisia. This emphasizes the urge to quantify the extent and severity of the resistance occurrence in *G. coranaria* in the most threatened wheat production areas of Tunisia. In this study, our interests were focused towards: (1) assessing the prevalence of *G. coranaria* and the main causes of its likely resistance occurrence through field surveys and farmers interviews; (2) determine the effects of *G. coranaria* densities on wheat yield losses; and (3) confirm the occurrence of resistance to ALS-inhibiting herbicide based on field trials and whole plant and dose–response assays.

## 2. Results

### 2.1. Effect of Farmers’ Practices on G. coronaria Occurrence and Associated Resistance Awareness

Surveys were conducted in northern country to assess the factors that led to the prevalence of *G. coronaria* in wheat crops. Based on interview data, 70% of the interviewed farmers affirmed the presence of *G. coronaria* in their fields and described it as a difficult weed to control (Table 1). Few farmers (33%) related the occurrence of *G. coronaria* to a possible herbicide resistance, while the others (67%) did not expect a causal relationship between the failure of the weed control and resistance occurrence in their fields. Cereal monoculture was adopted by a high proportion of interviewed farmers, the majority of them relying on conventional tillage (plow) and slightly delaying the date of sowing after mid-November (from 20th of November to 31st of December). The most used herbicides are those belonging to group B (principally sulfonylureas and triazolopyrimidines) or herbicide mixtures of groups O and B, while 30% of farmers used once-a-year herbicides of the group B, and 57% affirmed that they did not switch between herbicide MoAs. Herbicide mixtures and the appropriate setting of the sprayers were adopted by the majority of farmers, while full labeled doses and the use of a rescue treatment in spring were adopted by half of them (Table 1).

The results of chi-square test performed to determine the correlation between farmer’s practices and their suspicion of a possible resistance occurrence in *G. coronaria* showed that farmer’s awareness is significantly correlated with the practice of tillage and delay of the sowing date (Table 2). In fact, most of the farmers who are aware of a possible resistance occurrence in *G. coronaria* delay their cereal sowing until December as a major cultural practice to prevent high densities and competition with the crop. On the other hand, farmers practicing more plow as soil tillage were unaware of the occurrence of resistance in *G. coronaria*. Such unawareness may be attributed to reduced weed emergence and densities as a consequence of deep tillage; therefore, farmers do not consider the weed as a serious threat to their production.

Chemical managements, mainly herbicide rotation and herbicide frequency of use, were revealed as the causes of the *G. coronaria* severity occurrence in surveyed fields (Table 2). In fact, 79% of the growers did not use herbicide rotation, applying sulfonylureas and triazolopyrimidines (alone or mixed with group O) every year. However, less severity of *G. coronaria* was associated with more herbicide rotation and a reasonable use of herbicides of group B. Indeed, 50% of farmers with fewer problems to manage *G. coronaria* frequently alternate the use of herbicides of group B with herbicides belonging to other groups (i.e., group O, group A, or others).

The survey results showed that the widespread of the weed was correlated mainly to the extensive use of ALS-inhibiting herbicides. However, farmer’s awareness was expressed principally by cultural practices such as delaying the date of cereal sowing, while no action related to the herbicide use was undertaken. Farmers who did not suspect resistance occurrence, or even the spreading of the weed in their fields, attributed the failure of chemical control to several factors, including the use of an incorrect dose, the spraying setting, the absence of herbicide mixture, and the rescue herbicide application to control new emergence. Chi-square test was not able to relate *G. coronaria* spread to these factors, which may suggest resistance occurrence of *G. coronaria* to the ALS-inhibiting herbicides (group B) in the Bizerte region/Northen Tunisia.

### 2.2. Effect of G. coronaria Density on Wheat Yield

**Figure 1:**
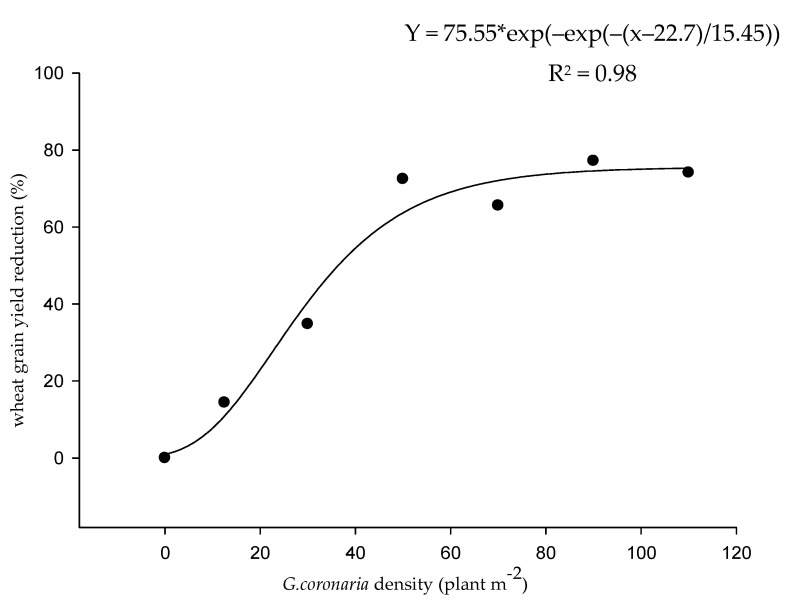
Effect of *G. coronaria* density on wheat grain yield reduction (%).

Different densities of *G. coronaria* were determined in the wheat fields and the mean infestation level was estimated at 30 plants m^−2^. The average levels of infestation per field varied between 9 (±5) plants m^−2^ to 83 (±88) plants m^−2^. All *G. coronaria* densities, except the 0–20 densities, significantly reduced the wheat grain yield compared to controls. Significant negative correlations (*p* = 0.01) were observed between *G. coronaria* density and wheat spikes densities (r = −0.922; Table 3) and between *G. coronaria* density and wheat grain yield (r = −0.939; Table 3). Data analysis revealed that increasing *G. coronaria* density from 20 plants m^−2^ to more than 100 plants m^−2^ resulted in a significant increase of wheat yield reduction (%) following a sigmoidal curve, reaching 75% for the higher density. Below the density of 20 plants m^−2^, nonsignificant yield losses (about 14%) were recorded (Figure 1).

### 2.3. Herbicide Sensitivity Assays and Confirmation of G. coronaria Resistance to ALS-Inhibiting Herbicides

#### 2.3.1. Herbicide Evaluation in Field

**Figure 2:**
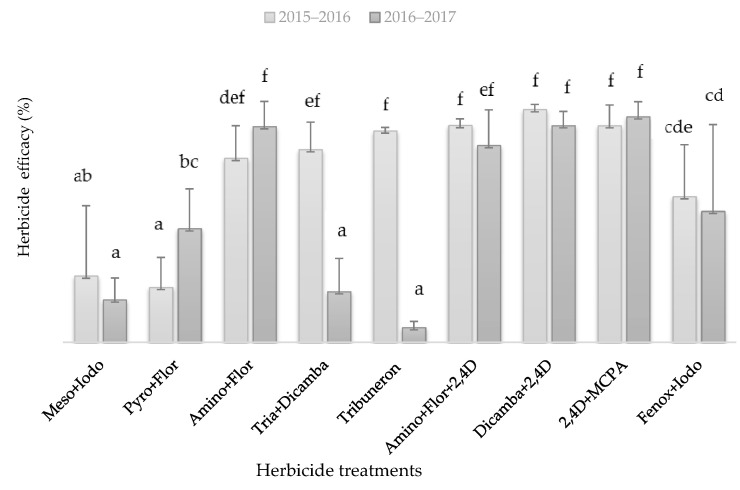
Efficacies of different herbicide treatments on *G. coronaria* dry weight reduction assessed in a field trial in two consecutive seasons (2015–2016 and 2016–2017). Different letters denote significantly differences between herbicides within a year according to the Duncan test (*p* = 0.05). Herbicide abbreviations; Meso + Iodo: mesosulfuron + iodosulfuron; Pyro + Flor: pyroxsulam + florasulam, Amino + Flor: aminopyralid + florasulam; Tria + Dicamba: triasulfuron + dicamba; Tribenuron: tribenuron−methyl; Amino + Flor + 2,4D: aminopyralid + florasulam + 2,4 D EHE ehe; Dicamba + 2,4D: dicamba + 2,4 D; Fenox + Iodo: fenoxaprop-p-ethyl + iodosulfuron.

In order to assess the efficacy of herbicides commonly used by farmers in the region, experiments were conducted in the same field for two consecutive years. In the first year (2015–2016), the high amount of annual rainfall was concentrated in spring, while the second experimental year (2016–2017) was characterized by a drier spring but a wet winter, associated with higher density of *G. coronaria* (data not shown). The two-way ANOVA results showed that herbicide efficacies depend significantly (*p* < 0.001) on the year (climatic conditions), the herbicide treatment and their interaction. According to the results represented in Figure 2, herbicide efficacies were lower during 2016–2017, especially in treatments with triasulfuron + dicamba and tribenuron-methyl, while the other herbicides, namely, mesosulfuron + iodosulfuron, pyroxsulam + florasulam, fenoxaprop-p-ethyl + iodosulfuron, were not effective to control *G. coronaria* in both years (efficacies ranged between 23% and 58%). The most effective treatments in reducing the weed biomass were with the auxinic herbicides, 2,4-D + MCPA and Dicamba + 2,4-D, with efficacies of 92% and 94%, respectively. The mixture of two modes of action, auxinic herbicides and ALS-inhibiting herbicides (triazolopyrimidines), such as aminopyralid + florasulam + 2,4 D EHE ehe and aminopyralid + florasulam treatments, were less effective compared to auxinic herbicides alone (efficacy around 85%).

#### 2.3.2. Sensitivity of G. coronaria to Two Sulfonylureas Herbicides

To investigate the spread of ALS-inhibiting herbicide resistance in the Bizerte region, two sulfonylureas herbicides (mesosulfuron + iodosulfuron and tribenuron-methyl) were tested at the field rate in 10 populations in natural conditions. All the plants from the reference population (S) were rated as sensitive for both herbicides and 100% of plant mortality was observed 10 days after treatment. Most tested populations were significantly able to survive both herbicides at field rate (Table 4). For the treatment with tribenuron-methyl herbicide, the percentage of weed survival ranged between 63% and 100%, and for eight out of the 10 populations, survivals were above 93%. A decrease of sensitivity was observed for all populations, except P3 and P9, when they were treated with mesosulfuron + iodosulfuron. The percentage of plant survival ranged between 59% and 89%. The population P3 was the only one able to fully survive both herbicide treatments.

**Figure 3:**
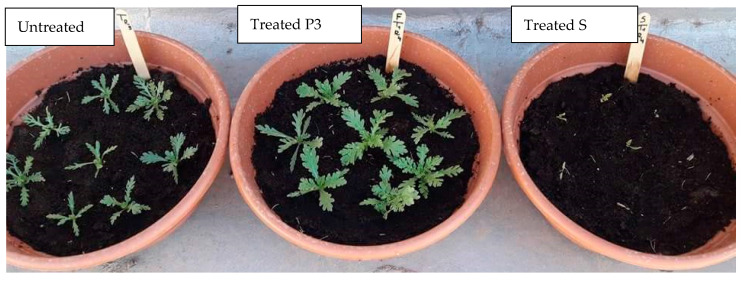
Growth differences among *G. coronaria* plants observed 10 days after tribenuron-methyl application at field rate. Right, untreated control; middle, treated P3 population; left, treated S population.

Overall, a clear shift towards the increase of dry biomass was observed for most populations after 28 days after treatment (DAT) (Figure 3). Considerable increases in dry biomass were observed in P3, P4, P5, and P6 populations after both herbicide applications, particularly associated with tribenuron-methyl, reaching 59% and 40% for P3 and P4, respectively. In contrast, plants from the P8 population showed a decrease in the dry biomass accumulation after both herbicides’ application, being more pronounced for the mesosulfuron + iodosulfurontreatment. The other populations (P1, P2, P7, P9, P10) behaved differently depending on the herbicide.

These results showed a decrease of sensitivity of *G. coronaria* to sulfonylureas chemical family, P3, P4, and P5 populations showed the highest ability to tolerate both herbicides, while P8 seemed to be the most sensitive population, especially for mesosulfuron + iodosulfuron herbicide. This ability to tolerate sulfonylureas herbicides was associated with a stimulation of dry biomass compared to nontreated plants.

#### 2.3.3. Dose–Response Experiments

**Figure 4:**
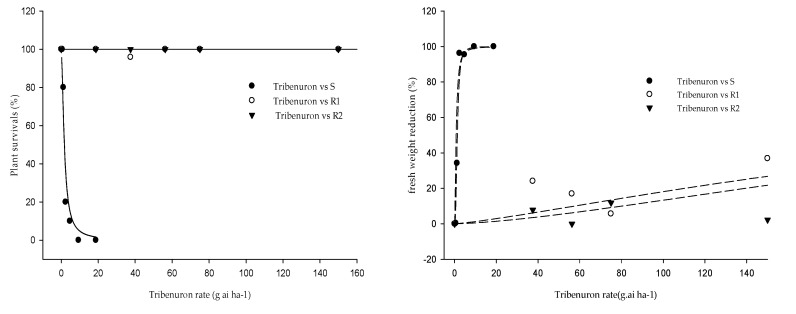
Effects of tribenuron-methyl applied at increasing rates on the plant survivals (%) and shoot fresh weights of sensitive (S) and resistant (R1 and R2) *G. coronaria* populations 21 days after treatment.

In order to estimate the level of resistance developed in two selected *G. coronaria* populations (P3 and P5 named here R1 and R2 respectively), dose–response experiments were conducted (Table 5 and Figure 4). The tested doses were not able to decrease the percentage of survival for both populations and no significant reduction in fresh weights were recorded in response to increasing doses of tribenuron-methyl, indicating that R1 and R2 were highly resistant to this herbicide compared to the S population. The ED50 and LD50 values exceeded the highest applied rate (300 g ha^−1^), which eventually resulted in failure to fit the sigmoidal model. The sensitive population responds to the increased doses, reaching 100% of mortality at a quarter of the field dose (9.38 g ha^−1^) and the ED50 and LD50 determined were 1.7 and 1.4 for weed survival and fresh weight, respectively.

## 3. Discussion

### 3.1. Glebionis coronaria Occurrence in Northern Tunisia

Field surveys were conducted in Northern Tunisia in the region of Bizerte where wheat crops are widely grown by farmers [4]. Results of the interviews made with the farmers revealed the reliance of growers on cereal monocropping under rainfed conditions, which may be attributed to the high price of the durum wheat in Tunisian market, thus making continuous wheat cropping the most adopted practice by farmers of the region. However, such cultural practice usually selects for weeds species having phenological and physiological similarities to the crop [18,19]. This may explain the abundance of *G. coronoria*, among others weed species in the fields continuously sown with winter wheat. The ALS-inhibiting herbicides (group B) or mixed herbicides with two MoAs (ALS inhibitors and auxinic herbicides) were frequently used by farmers to control weed flora associated to winter wheat. The abundance of rigid ryegrass (*Lolium Rigidum* Gaud.) another problematic weed in the same fields [20], may explain in part the continuous use of the ALS-inhibiting herbicides. The lack of herbicide rotation and the high frequency of the use of herbicides of the group B were highly correlated with *G. coronaria* occurrence in our survey. Previous studies focusing on the rotation of the mode of action (MoA) within different cropping systems to delay weed selection, showed that the frequency of herbicide use was the most important factor to explain the dominance of weed species and the occurrence of herbicide resistance [10,21]. Our study showed that few farmers are aware of the possible occurrence of resistance in *G. coranaria*. Practically, this awareness was associated with late sowing of wheat, which increases the suppression of emerging weed seedlings while preparing the seedbed for sowing [22]. On the other hand, unaware farmers were those usually adopting conventional tillage. In fact, the deep soil disturbance resulting from plowing decreases the germination and the emergence rates of low dormant weed seeds [18], leading to a decrease in the spreading of weeds throughout the field. Despite the effect of soil tillage in reducing weed infestations, conventional tillage used in monocropping systems still remains ineffective to avoid the risk of the occurrence of herbicide resistance [19].

Surveys conducted in our study also showed high levels of infestations by *G. coronaria*. The fitting adopted for wheat and *G. coronaria* competition was sigmoidal. Previous studies reported that the gentle initial slope of the sigmoidal fitting would imply high weed control thresholds [23,24]. In our study, a density of more than 20 plants m^−2^ could be a critical density since it is associated with significant yield losses ranging between 5% to 20% as reported previously [25,26]. Several studies related to the competitive ability of broad-leaved weeds in wheat pointed out the negative correlation between crop yield and weed density [27,28,29]). For a high density of 80 plants m^−2^, *Galium aparine*, a troublesome weed in winter wheat, reduced the grain yield by 31.5% [28], reaching 57% in other studies in England [27], Our results indicated that *G. coronaria* caused 72% of yield losses for the same density (based on sigmoidal curve equation); thus, *G. coronaria* is likely more competitive. In contrast, the competitive ability of *G. coronaria* was similar to *Sinapis arvensis* [30] for middle density (30 plants m^−2^), causing 34% and 36% of wheat yield losses, respectively. Similarly, for *Papaver rhoeas*, wheat yields decreased up to 32% [31]. In conclusion, *G. coronaria* competes vigorously with winter wheat, like previous known troublesome weeds. In fact, weeds from the Asteraceae family are very competitive due to their vigorous growth and potential to exploit available resources [29,32]. This competitive ability of *G. coronaria* and it is associated wheat yield penalty emphasize the need for correct weed management decisions. In addition, the high levels of infestations recorded in this study may increase the probability of recurrent selection of resistance in *G. coronaria* with the use of the same MoA [10,11,33].

### 3.2. Confirmation of *G. coronaria* Resistance to ALS-Inhibiting Herbicides

This study showed low efficacies of sulfonylurea herbicides (tribenuron-methyl and mesosulfuron + iodosulfuron) to control *G. coronaria*. The pot experiments using these herbicides confirmed previous findings in field conditions in ten populations across the Bizerte region in northern Tunisia, which may suggest that resistance to sulfonylurea’s chemical family is spread across the region. In the dose–response experiments with tribenuron-methyl, two populations were confirmed as resistant to sulfonylureas. Both R1 and R2 were highly resistant to tribenuron-methyl, and all plants survived 16 times the recommended field dose, as reported in previous studies in *G. coronaria* [17] or in others broadleaf species [34], these include *Descurainia sophia* [35], *Lamium amplexicaule* [36], *Papaver rhoeas* [37], *Conyza sumatrensis* [38], *Diplotaxis erucoides*, and *Erucaria hispanica* [39]. Finally, in field trials, triazolopyrimidines (pyroxsulam + florasulam) control levels were limited or even ineffective, which suggests a possible cross resistance to this ALS family, as reported in other weed species [36].

All studied populations came from fields treated for a long time with ALS-inhibiting herbicides to control grasses and dicotyledonous weeds in winter wheat. According to this study, putative populations resistant to ALS inhibitors could be widely distributed in Northern Tunisia. Similar results were found for *D. sophia* in China [34], as well for *L. rigidum* in Tunisia [40], another troublesome weed species in the region, which behaved similarly and had evolved resistance in 87% of tested populations [41]. At field rate, at least for one of the two sulfonylureas tested, the dry weight increased compared to nontreated plants in most populations which suggest a hormetic effect induced in *G. coronaria* resistant plants. Researchers hypothesized that highly resistant individuals may be especially responsive to hormesis. The dose–response experiment for tribenuron supports this hypothesis [42].

This study represents the first report in Tunisia and second case worldwide of resistance to ALS-inhibiting herbicides in *G. coronaria*. The high levels of resistance pointed to a target site alteration of the *ALS* gene encoding the herbicide target ALS enzyme. In the future, molecular studies will be crucial to confirm the presence in this weed of TSR mechanisms to the ALS-inhibiting herbicides.

## 4. Materials and Methods

### 4.1. Field Surveys and Farmer Interviews: Occurrence and Resistance Awareness

**Figure 5:**
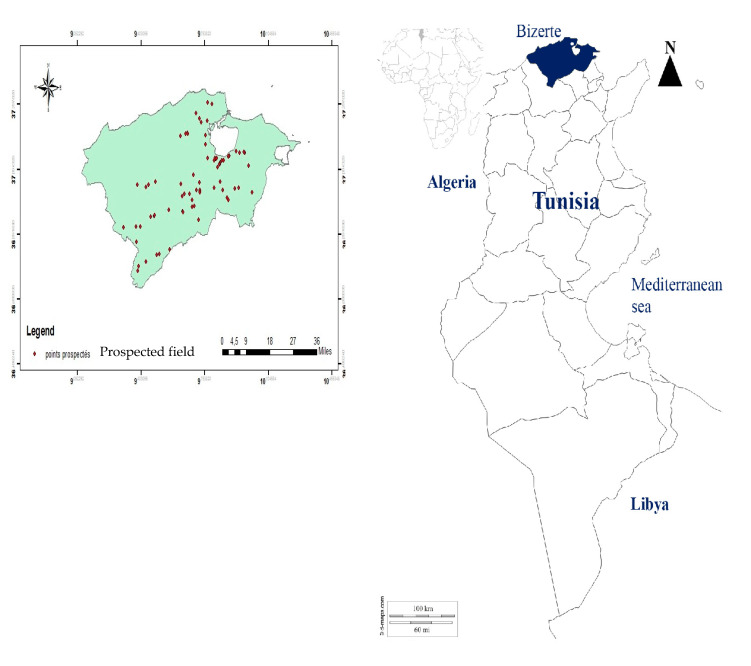
The investigated fields of durum wheat *(Triticum turgidum* L.) in Bizerte region in Northern Tunisia.

In order to determine the main management practices leading to *G. coronaria* occurrence and the associated farmers’ awareness of herbicide resistance evolution, a survey was conducted over a period of three years (from 2016 to 2018) in the region of Bizerte in Northern Tunisia (37°16′28.603′′ N, 9°51′45.806′′ E, 3750 km^2^). The field surveys were conducted late in the growing season after the last herbicide treatment but before harvest started. Across the region, seventy-six infested durum wheat fields were located (Figure 5).

The analysis focused on 46 growers who had observed reduced herbicide efficacy to control *G. coronaria* (especially with ALS-inhibiting herbicides), over one to five years period as an average. The main cultural practices and the chemical management strategy for at least the last three years were recorded for each interviewed grower.

The investigated factors were crop rotation, tillage, sowing date, herbicide MoAs, frequency of B-group use, herbicide rotation, full labeled rate, herbicide mixture, sprayer setting, and rescue treatment (the use of an extra herbicide application in spring, especially for *G. coronaria*). Categories within each factor were adjusted and the frequency distribution of cases (%) within each category of analyzed factors were calculated. The grower awareness and the *G. coronaria* occurrence represent the categorical response using “yes” (farmer awareness of resistance/serious occurrence of *G. coronaria*) and “no” (farmer non awareness of resistance/controlled occurrence *G. coronaria*).

### 4.2. The effects of G. coronaria Densities on Durum Wheat Yield Losses

Across the surveyed fields, 10 winter wheat *(Triticum turgidum* L.) fields naturally infested by *G. coronaria* were chosen to assess its density effect on wheat yield. In all fields, growers used almost the same agronomic practices and *G. coronaria* was the main weed present after herbicide treatments. Sampling of *G. coronaria* and wheat was done at wheat maturity in June 2018 using a systematic selection [43]. From a fixed area of 50 m^2^ (only with *G. coronaria*) in each field, five quadrates of 1 m^2^ were sampled in a zigzag arrangement. Quadrates without *G. coronaria* were used as controls from each field. Seven pure stand density classes (plant m^−2^) of *G. coronaria* (0, 4–20, 20–40, 40–60, 60–80, 80–100, >100) were defined to simplify the analysis. Every density was represented by at least three observations. From each field, wheat and *G. coronaria* were hand harvested. Then, the number of *G. coronaria* plants m^−2^, average weight of *G. coronaria* in g m^−2^, number of wheat spikes m^2^ and wheat grain yield (g m^−2^) were determined for each sample. Wheat yield losses was expressed by the grain yield losses in each field and for each weed density. The observed yield loss (%) across densities compared to weed-free control was calculated as

$$\mathrm{Grain}\;\mathrm{yield}\;\mathrm{losses}\;(\%)=\frac{\mathrm{Ywf}-\mathrm{Yw}}{\mathrm{Ywf}}\;*100$$

where Ywf and Yw represent wheat yields in weed-free control and weedy quadrats, respectively.

### 4.3. Herbicide Performance to Control G. coronaria

#### 4.3.1. Field Experiments

To evaluate the efficacy of the most common postemergence herbicides used by Tunisian farmers to control *G. coronaria*, field experiments were conducted during two seasons (2016 and 2017) at Fritissa, Mateur-Bizerte (37°1′50.91′′ N, 9°42′26.92′′ E) under rainfed conditions (Table 6). The soil was clay-loam in texture, relatively rich on organic matter (2.3%) with alkaline reaction (pH of 8.5). This field was chosen owing to (i) *G. coronaria* dominance, natural occurrence and high infestation (143 plant/m^2^ ± 82), (ii) the monoculture cultivation system of durum wheat adopted by farmers for eight years, (ii) the reduced efficiency of sulfonylurea herbicides in controlling *G. coronaria* as reported by farmers. The herbicides were applied at the recommended doses (Table 7) in mid-February corresponding to the vegetative stage of *G. coronaria* using a backpack sprayer calibrated to deliver 200 L ha^−1^ at a pressure of 3 KPa.

The experiment was performed in a randomized complete block design (RCBD) with four replicates per herbicide treatment. Each experimental unit covered an area of 30 m^2^ (5 m × 6 m). Four untreated plots were used as control. The efficacies of herbicides on *G. coronaria* was evaluated at 40 days after treatment (DAT) based on weed dry biomass reduction. Above-ground weed plants were harvested from three 1 m^−2^ quadrats randomly selected in each treated plot and untreated ones, and then oven-dried for 48 h at 70 °C before weight measurements.

#### 4.3.2. Evaluation of Sulfonylureas in Pots

To evaluate the spread of *G. coronaria* in the region of Bizerte, ten population (the field population named P3 plus nine other populations) were collected randomly from surveyed wheat fields in summer 2016 and screened for their sensitivity to ALS-inhibiting herbicides. Seeds were harvested from 30 plants per field, mixed together, placed in unsealed paper bags and stored at room temperature [44]. An additional susceptible (S) population was collected from the roadsides of the National Agronomic Institute of Tunisia (36°49′49.01′′ N; 10°11′02.07′′ E). This population has never been treated with any ALS-inhibiting herbicide.

Prior to starting the whole plant assay, *G. coronaria* seeds were scarified and pregerminated, then transplanted individually in plastic trays cell (0.7 mm) filled with commercial substrate and grown under natural conditions. At the two-leaf stage, homogeneous plants were selected to be transplanted in pots (60 cm of diameter and 20 cm of high) with a density of 8 plants per pot. Two sulfonylureas herbicides (Tribenuron-methyl and mesosulfuron + iodosulfuron) were applied at the four-leaf stage and the recommended doses were used. The number of surviving plants and the average of dry biomass per plant were recorded at 28 DAT. Four replications per treatment and per population were designed as a RCBD. Weight reduction was calculated as a percentage of the untreated control as following

$$\mathrm{Dry}\;\mathrm{weight}\;\mathrm{reduction}\;(\%)=\;\;\frac{\left(\mathrm{DWc}-\mathrm{DWt}\right)}{\mathrm{DWc}}*100$$

where Wc is the average weight of nontreated plants and Wt is the average weight in treated plants.

#### 4.3.3. Tribenuron-Methyl Dose–Response Assay

For this experiment, the two most resistant populations (R1 and R2) and the susceptible one (S) described in the Section 4.3.2 were used. *G. coronaria* plants were grown as above. At the cotyledon stage, two *G. coronaria* seedlings were transplanted per pot. At the 2–4-leaf stage, plants were sprayed with tribenuron-methyl, at 0, 18.75, 37.5, 56.25, 75, 150, 300 g a.i. ha^−1^ for R1 and R2 and 0, 0.29, 0.59, 1.17, 2.34, 4.69, 9.37 g a.i. ha^−1^ for the S one. The whole experiment was arranged in a RCBD design and ten pots (replication) were used for each herbicide dose. A precision bench sprayer with two Hardi ISO LD-110-02 flat fan 110° opening nozzles operating at a forward speed of 0.9 m s^−1^, 50 cm above plants, 200 l ha^−1^, and at a pressure of 215 kPa were used to apply the herbicide. For each dose of tribenuron-methyl and each population, the number of survivors and the fresh weight reduction were determined three weeks after treatment. Fresh weight reduction was calculated using the following equation

$$\mathrm{Fresh}\;\mathrm{weight}\;\mathrm{reduction}\;(\%)=\;\frac{\mathrm{FWc}-\mathrm{FWt}}{\mathrm{FWc}}*100$$

where FWc is the average weight of nontreated plants and Wt is the average weight in treated plants at dose x.

### 4.4. Statistical Analysis

For the survey and interview results, answers were considered as factors and categories within each factor were adjusted. Frequencies were calculated by SPSS (IBM SPSS statistics 20) contingency tables and the correlations between cultural and management practices (the explanatory variables) and grower awareness and *G. coronaria* occurrence (the response variables) were analyzed using the Pearson Chi-square test at 90% confidence level (*p*-value = 0.100). One-way ANOVA were performed with SPSS software and means were compared using the Duncan post hoc pairwise test (*p*-value = 0.05) to analyze the *G. coronaria* density effect on grain yield reduction. Pearson correlation coefficients “r” were used to assess the correlation between *G. coronaria* (density m^−2^ and dry biomass m^−2^) and wheat parameters (number of wheat spikes m^−2^, wheat grain yield m^−2^).

For the field experiment, a two-way ANOVA was conducted to analyze the effect of two factors, herbicide treatment (T) and year (Y) and their interaction (T × Y), on the weed control. One way ANOVA was conducted for both tribenuron-methyl and mesosulfuron + iodosulfuron treatments to assess their efficacies on survivals and dry biomass of *G. coronaraia* populations. Nonlinear regression models were carried out for both *G. coronaria* density effect on wheat grain yield and dose–response experiment using Sigmaplot 11.0 (Systat Software, San Jose, CA, USA). Sigmoidal fitting (Gompertz equation with three parameters) was used to correlate the *G. coronaria* density and the wheat yield losses following the Equation (1):

(1) $$y\left(\%\right)=a*e^{-e\left(-\frac{x-x_{0}}{b}\right)}$$

where a = the upper limit of the curve, b = the lower limit of the curve, x_0_ = the inflexion point, x is the density of *G. coronaria* and y is the grain wheat yield losses. Tribenuron doses causing 50% of *G. coronaria* growth reduction (EC50) and mortality (LD50) in resistant and susceptible populations were determined based on the following four-parameter log-logistic equation (Equation (2))

(2) $$y=c+\frac{\left(D-C\right)}{1+{\left(\frac{x}{EC50}\right)}^{-b}}$$

c = the lower limit adjusted to 0, d = the upper limit adjusted to 100 and b = the slope at the EC50 or LD50, x was the independent variable (dose of tribenuron applied (g a.i.ha^−1^),) and y was the percentage of survivals/fresh weight reduction for each population.

## 5. Conclusions

Based on the results of this study, herbicide resistance in *G. coronaria* has evolved as a result of a decrease of the efficacy of ALS-inhibiting herbicides, mainly due to wheat monocropping and high frequencies of herbicide treatments with the same MoA. Field and pot experiments showed that the presence of resistance to sulfonylureas is widespread in the region of Bizerte in Northern Tunisia, which was confirmed for tribenuron-methyl in two populations. However, farmers were not aware of this threat; therefore, more proactive efforts are required to increase both the responsiveness of farmers to the evolution of herbicide resistance in this weed species and the implementation of integrated weed management strategies based on alternative MoA (i.e., auxinic herbicides and others) and adequate cultural and management practices to mitigate the occurrence of *G. coronaria* resistance.

## Figures and Tables

**Table 1:** Frequency distribution of cases (%) within each category of analyzed factors (cultural and management factors) and frequency distribution of grower resistance awareness (%) for *G. coronaria* (%) are shown in each category.

Factors and Categories	Frequency Distribution of Cases (%)	Grower Resistance Awareness ^†^ (%)		*G. coronaria* Occurrence ^††^ (%)	
		Yes	No	Yes	No
	Total (100%)	32.6	67.4	69.6	30.4
**Crop rotation**					
Monoculture	60.9	46.7	67.7	59.4	64.3
Biennial	30.4	40.0	25.8	28.1	35.7
Triennial	8.7	13.3	6.5	12.5	0.0
**Tillage**					
Plow	74.4	54.5	82.1	73.1	76.9
Reduced	25.6	45.5	17.9	26.9	23.1
**Sowing Date**					
Early / Middle	43.5	26.7	51.6	43.8	42.9
Late	56.5	73.3	48.4	56.2	57.1
**herbicide groups**					
group B	30.4	42.9	25.8	31.3	28.6
group O	4.4	7.1	3.2	6.2	0.0
group A	17.4	7.1	22.6	18.8	14.3
group O+B	41.3	35.7	41.9	40.6	42.9
others	6.5	7.2	6.5	3.1	14.2
**Frequency of B-group uses**					
0/3	15.2	6.7	19.4	15.6	14.3
1/3	28.3	40.0	22.6	28.1	28.6
2/3	26.1	20.0	29.0	15.7	50.0
3/3	30.4	33.3	29.0	40.6	7.1
**Herbicide rotation**					
Yes	43.5	46.7	42.0	31.2	71.4
No	56.5	53.3	58.0	68.8	28.6
**Labeled dose**					
Yes	58.7	53.3	61.3	59.4	57.1
No	41.3	46.7	38.7	40.6	42.9
**Herbicide mixture**					
Yes	60.9	46.7	67.7	59.4	64.3
No	39.1	53.3	32.3	40.6	35.7
**Sprayer setting**					
Yes	82.6	93.3	77.4	78.1	92.9
No	17.4	6.7	22.6	21.9	7.1
**Rescue treatment**					
Yes	56.5	53.3	58.1	56.2	57.1
No	43.5	46.7	41.9	43.8	42.9

^†^ Grower resistance occurrence: refers to the grower suspicion of potential resistance occurrence in his filed; ^††^ *G. coronaria* occurrence: refers to the severity of the weed prevalence in each field based on the grower observation.

**Table 2:** Chi-Square test for dependency of grower awareness and *G. coronaria* occurrence to cultural and management practices in wheat field (Bizerte region).

Response Variables	Factors	Pearson Chi-Square		
		Value	df	Asymptote Sign. (2-Side)
**Resistance awareness (%)**	Tillage	3.155	1	0.076 *
	Sowing Date	2.560	1	0.110 *
***G. coronaria*occurrence (%)**	Herbicide rotation	6.398	1	0.011 **
	Frequency of B-group uses	8.011	3	0.045 **

* Factor significant at 90% confidence level (*p*-value ≤ 0.1); ** Factor significant at 95% confidence level (*p*-value ≤ 0.05).

**Table 3:** Pearson’s correlation matrix for *G. coronaria* traits (density in plants m^−2^, average plant dry weigh in g m^−2^) and wheat yield components (spikes density in number m^−2^, grain yield in g m^−2^).

	Density	Dry Weigh	Spikes	Grain Yield
**Density**	1	**	**	**
**Dry weigh**	0.963	1	**	**
**Spikes**	−0.922	−0.925	1	**
**Grain yield**	−0.939	−0.932	0.971	1

** Correlation is significant at the 0.01 level (two-tailed).

**Table 4:** *Glebionis coronaria* survival and average dry weight reduction/stimulation at 28 days after treatment (DAT) after two sulfonylureas herbicides application.

Population	Survivals (%)		Average Dry Weight (g), Increase Rate (IR) and Decrease Rate (%)			
	Tribenuron-Methyl	Mesosulfuron + Iodosulfuron	Tribenuron-Methyl		Mesosulfuron + Iodosulfuron	
			**Dry Weight**	**IR/DR**	**Dry Weight**	**IR/DR**
P1 bcd	79.2	79.2	5.03	IR = 9.7	4.07	DR = 11.4
P2 bc	93.8	59.4	6.75	IR = 47.2	2.57	DR = 43.9
P3 a	100	100	7.30	IR = 59.0	4.94	IR = 29.4
P4 ab	100	87.5	6.44	IR = 40.3	5.58	IR = 21.5
P5 ab	96.9	93.8	5.58	IR = 21.6	5.52	IR = 20.3
P6 bc	100	87.5	5.31	IR = 15.8	4.64	IR = 1.1
P7 bc	96.9	87.5	5.56	IR = 21.1	4.58	DR = 0.2
P8 d	90.6	59.4	4.53	DR = 1.3	1.75	DR = 61.9
P9 bc	62.5	90.6	4.07	DR = 11.3	5.84	IR = 27.3
P10 cd	93.8	68.8	5.26	IR = 14.6	2.25	DR = 51.0
PS ^†^	0.0	0.0	0.0	-	0.0	-
CNT ^††^	100	100	4.59	-	4.59	-
**ANOVA**	Df	F value		Df	F value	
**Population**	1	15.567 ***		1	17.764 ***	
**Treatment**	9	5.020 ***		9	4.246 ***	
**Interaction**	9	3.622 *		9	2.901 *	

^†^ PS: susceptible population, ^††^ CNT: nontreated control, The F values are shown and the symbols indicate statistical significance (*, *p* = 0.05; ***, *p* = 0.001), Population with different superscript letters are significantly different classes according to the Duncan test (*p* = 0.05) using average dry weight reduction/stimulation.

**Table 5:** Parameters of the log-logistic equations used to calculate the tribenuron-methyl rates required for 50% reductions in fresh weight (ED50) and percent survival (LD50) expressed as the percentage of the mean untreated control of the *G. coronaria* populations.

	Population	ED50/LD50	b	*p*	RI50
*% Survival*	R1	>300	-	-	>300
	R2	>300	-	-	>300
	S	1.7	−2.4	<0.001	-
*% Fresh weight*	R1	>300	-	-	>300
	R2	>300	-	-	>300
	S	1.4	2.7	<0.001	-

**Table 6:** Seasonal precipitation and temperature of the two years of field experiment in Fritissa/Mateur, Bizerte region.

	2015–2016			2016–2017		
Season	Cumulative Precipitation (mm)	T_Max_ (C)	T_Min_ (C)	Cumulative Precipitation (mm)	T_Max_ (C)	T_Min_ (C)
**Autumn**	67.1	19	15	92.7	20	15
**Winter**	85.2	16	12	251.3	15	11
**Spring**	147.3	19	13	51.7	20	14
**Summer**	15.8	26	19	12	28	21
Sum/average *	315.4	20	14.75	407.7	20.75	15.25

* Sums for precipitation; average values for Tmax, Tmin.

**Table 7:** Herbicide treatments tested in field experiments.

Active Ingredient	Group (Chemical Family *)	Amount of Active Ingredient	Applied Doses
**mesosulfuron + iodosulfuron**	B (S)	30 g Kg^−1^ + 30 g Kg^−1^	330 g ha^−1^
**pyroxsulam + florasulam**	B (Tri)	70.8 g Kg^−1^ + 14.2 g kg^−1^	320 g ha^−1^
**aminopyralid + florasulam**	O + B (Tri)	35.5% + 15%	33 g ha^−1^
**triasulfuron + dicamba**	B (S) + O	41 g Kg^−1^ +659 g Kg^−1^	180 g ha^−1^
**tribenuron-methyl**	B (S)	75%	25 g ha^−1^
**aminopyralid + florasulam + 2,4 D EHE ehe**	O + B (Tri)	300 g L^−1^ + 6,25 g L^−1^	0.6 L ha^−1^
**dicamba + 2,4 D**	O	120 g L^−1^ + 344 g L^−1^	0.8 L ha^−1^
**2,4-D + MCPA**	O	345 g L^−1^ + 345 g L^−1^	1.5 L ha^−1^
**fenoxaprop-p-ethyl + iodosulfuron**	A + B (S)	64 g L^−1^ + 8 g L^−1^	1 L ha^−1^

* S: sulfonylureas; Tri: triazolopyrimidines.
